# Atomic-Scale Mechanisms and Damage Suppression in Nanometric Cutting of Polycrystalline Copper: A Molecular Dynamics Study

**DOI:** 10.3390/nano16090564

**Published:** 2026-05-02

**Authors:** Yang Li, Peng Fu, Huan Gu, Shulin Liang, Lin Li, Hao Jiang, Yuan Hong, Zhan Li, Lei Lu, Rongrong Tang, Zhuo Li, Liqi Li

**Affiliations:** 1 CSSC Digital Information Technology Co., Ltd., Jiangsu Automation Research Institute, Lianyungang 222006, China; 2 Beijing Institute of Mechanical Equipment, Beijing 100000, China; 3 College of Mechanical and Vehicle Engineering, Hunan University, Changsha 410082, China; 4 Institute of Manufacturing Engineering, Huaqiao University, Xiamen 361021, China

**Keywords:** polycrystalline copper, molecular dynamics simulation, nanometric cutting, subsurface damage, diamond tool wear

## Abstract

Molecular dynamics simulations were performed to investigate the nanometric cutting of polycrystalline oxygen-free copper using a single-crystal diamond tool. The effects of grain size, tool geometry (rake angle and edge radius), cutting speed, and ambient temperature on atomic migration, dislocation activity, and tool wear were systematically analyzed. The results indicate that material removal is dominated by cutting-induced amorphization and the formation of hcp-coordinated defect structures, while dislocation activity governs plastic deformation and cutting force fluctuations. A damaged subsurface layer, composed of amorphous structures, hcp-coordinated defects, and residual dislocations, is formed beneath the machined surface. Increasing grain size reduces grain-boundary-induced stress concentration and suppresses subsurface damage. A larger rake angle facilitates chip removal and reduces damage, whereas a larger edge radius intensifies dislocation activity and amorphization. Higher cutting speeds reduce lattice distortion and subsurface damage but increase stress concentration on the tool. Elevated temperature enhances atomic mobility, promoting amorphization and subsurface deformation while accelerating tool wear. These findings provide insight into the nanometric cutting behavior of polycrystalline copper and offer guidance for optimizing process parameters to improve surface integrity and tool life.

## 1. Introduction

Benefiting from its extremely high purity (copper content > 99.5%) and ultra-low oxygen content (oxygen content < 0.001%), polycrystalline copper (oxygen-free copper, OFC) exhibits outstanding electrical and thermal conductivity, making it one of the most important conductive materials for electronic components, second only to silver [1,2]. In addition, polycrystalline copper possesses excellent ductility, plasticity, and machinability, enabling the fabrication of complex geometries such as fine wires, thin strips, and vacuum components [3]. Moreover, when heated in high-vacuum or reducing atmospheres, OFC does not suffer from hydrogen embrittlement, which commonly occurs in conventional copper materials, thereby ensuring long-term reliability under harsh service environments [4,5]. Owing to these superior material properties, polycrystalline copper has found extensive applications in electronic components that require high electrical and thermal conductivity as well as excellent stability in vacuum environments, such as high-frequency electronic devices, vacuum-sealed components, and nuclear reactor systems [6].

Applications in precision electronic devices impose extremely stringent requirements on surface quality, typically with surface roughness values below Ra < 10 nm. To meet these demands for high surface integrity and dimensional accuracy, a variety of mature precision machining technologies for polycrystalline copper have been developed [7]. Conventional precision cutting and micro-cutting techniques remain the primary processing methods, where the use of high-speed spindles and diamond or ultra-hard alloy tools, combined with small depths of cut and low feed rates, effectively suppresses burr formation and achieves low surface roughness [8,9]. Ding et al. [10] investigated diamond micro-tool cutting of polycrystalline copper and analyzed the effects of crystallographic parameters and process parameters on cutting forces, surface roughness, and chip morphology. Their results demonstrated that at ultra-low cutting depths (<0.4 μm) and extremely small feed rates, tool damage can be significantly reduced while burr formation is minimized or even eliminated, leading to substantial improvements in machining quality. Single-point diamond turning (SPDT) can achieve nanometric surface roughness under certain conditions and is suitable for producing ultra-smooth polycrystalline copper surfaces; however, its performance is strongly influenced by grain size and crystallographic anisotropy [11]. Precision grinding and polishing technologies, which rely on fine abrasive particles for stable material removal, can effectively improve surface morphology and are often combined with chemical mechanical polishing (CMP) [12] or mechanical–chemical polishing (MCP) [13] to reduce subsurface damage. Overall, precision machining of polycrystalline copper is evolving toward multi-process integration and ultra-precision manufacturing to meet the increasingly stringent functional and reliability requirements of high-end manufacturing industries.

Due to variations in grain orientation and the high plastic deformability of polycrystalline copper, machining processes are prone to issues such as tool adhesion and surface tearing, placing higher demands on material properties, tool geometry, and cutting parameters. Zhang et al. [14] investigated the influence of crystallographic planes on surface morphology and machining quality during SPDT of single-crystal copper, demonstrating that the (100) plane yields the best surface quality. Chen et al. [15] revealed that grain orientation and grain size significantly affect cutting forces, chip morphology, and surface defects during SPDT of polycrystalline copper and showed that increasing cutting speed can suppress subsurface defects induced by grain orientation differences. Although SPDT enables mirror-finish machining of polycrystalline copper, limitations in experimental scale and characterization techniques hinder a clear understanding of the effects of grain parameters, process parameters, and tool geometry on cutting mechanisms and surface integrity at nanometric depths.

Molecular dynamics (MD) simulation, as a powerful numerical approach for revealing material deformation and removal mechanisms at the atomic scale, has attracted increasing attention in recent years for investigating precision machining mechanisms of polycrystalline copper [16,17,18]. Considering the random grain orientations and complex grain boundary distributions in polycrystalline copper, researchers have constructed realistic polycrystalline models with representative grain sizes and orientations to analyze atomic motion behaviors and lattice deformation mechanisms during nanometric cutting. Zhou et al. [19] employed MD simulations to study the effects of cutting depth, tool geometry, and grain orientation on nanometric cutting mechanisms of polycrystalline copper and analyzed the evolution of dislocations in the cutting deformation zone. Liu et al. [20] investigated the formation of grain boundary steps during nanometric cutting of polycrystalline copper and revealed that sub-grains with transitional crystallographic orientations form at grain boundaries due to tool plowing and crystal rotation effects, significantly influencing machining quality. Ranjan et al. [21] explored the influence of abrasive particle size and cutting speed on the shear slip and dislocation behavior in the cutting deformation zone of polycrystalline copper by MD simulation. The results showed that increasing abrasive particle size and cutting speed leads to a higher interaction force at the tool–workpiece interface, resulting in intensified shear slip, enhanced dislocation motion, and an increased material removal rate. Sharma et al. [22] studied the dislocation evolution behavior in the cutting deformation zone during the nano-cutting process of single-crystal copper by MD simulation and analyzed the influence of anisotropy on the cutting mechanism of single-crystal copper. The results showed that {111} plane<110>crystal direction was the least difficult to cut, and {110} plane<001>crystal direction was the most difficult to cut. Although existing MD simulation studies have deeply revealed the lattice evolution and dislocation motion during the nanoscale processing of polycrystalline copper, there is still no systematic analysis of the comprehensive impact mechanism of grain size, tool geometry, and process parameters on atomic displacement, lattice defect evolution, phase transformation, and damage suppression of the subsurface of polycrystalline copper processing.

In this study, an MD simulation model of single-crystal diamond tool cutting polycrystalline copper is established to investigate nanometric cutting mechanisms and dislocation evolution behaviors. By systematically varying material parameters, tool geometries, and process parameters, the effects of grain size, tool rake angle, tool edge radius, cutting speed, and ambient temperature on nanometric cutting mechanisms and machining damage of polycrystalline copper are revealed at the atomic scale.

While previous molecular dynamics studies on nanometric cutting of copper and other FCC metals have provided valuable insights into chip formation, dislocation activity, and cutting forces, most focus on single-crystal systems or consider individual parameters in isolation [23,24]. In addition, the role of grain boundaries and microstructural heterogeneity in polycrystalline materials, as well as their interactions with tool geometry and cutting conditions, remains not yet fully understood. In this context, the present study examines polycrystalline oxygen-free copper and investigates the combined effects of grain size, tool geometry, cutting speed, and ambient temperature within a unified simulation framework. Particular attention is given to correlating atomic-scale features—such as defect evolution, hcp-like local structures, and amorphization—with macroscopic responses, including subsurface damage, cutting forces, and tool wear. The results provide further insight into the nanometric cutting behavior of polycrystalline FCC metals.

## 2. Simulation Methodology

Molecular dynamics simulations of single-crystal diamond tool cutting polycrystalline copper were performed using the Large-scale Atomic/Molecular Massively Parallel Simulator (LAMMPS) [25], and the simulation results were visualized and analyzed using OVITO.

### 2.1. Material Models

**Figure 1:**
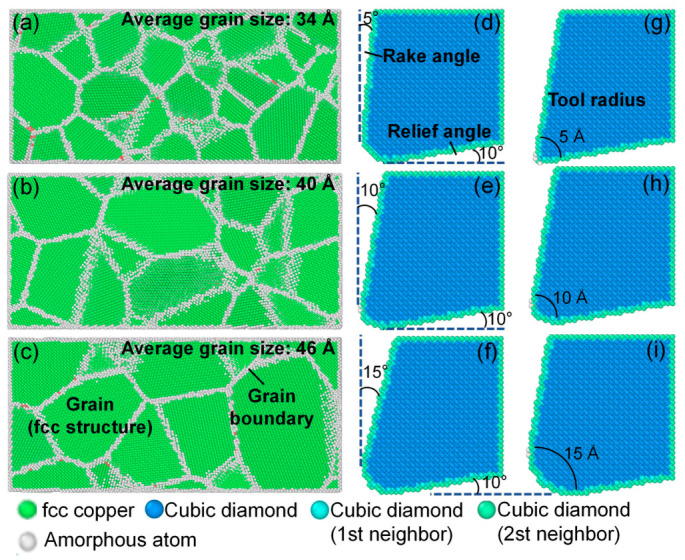
Polycrystalline copper material models and diamond tool models. Polycrystalline copper models with different grain sizes: (**a**) average grain size: 34 Å; (**b**) average grain size: 40 Å; and (**c**) average grain size: 46 Å. Diamond tool models with different geometrical parameters: (**d**) rake angle 5°; (**e**) rake angle 10°; (**f**) rake angle 15°; (**g**) edge radius 5 Å; (**h**) edge radius 10 Å; and (**i**) edge radius 15 Å.

Given the extremely low oxygen content in polycrystalline copper (mass fraction < 0.1%), the material model can be reasonably approximated as a single-element polycrystalline copper system composed solely of Cu atoms. Polycrystalline copper models were constructed using Atomsk [26], as shown in Figure 1. The model dimensions were 260 × 120 × 120 Å (length × width × height) and consisted of 40 copper grains with randomly distributed crystallographic orientations. Copper atoms within each grain follow a face-centered cubic (fcc) lattice structure with a lattice constant of 3.615 Å.

To investigate the influence of grain size on cutting mechanisms, three polycrystalline copper models with different average grain sizes were constructed, as shown in Figure 1a–c, with average grain sizes of 34 Å, 40 Å, and 46 Å, respectively. Due to computational limitations inherent to molecular dynamics simulations, the grain sizes considered in this study (34–46 Å) are smaller than those in practical polycrystalline oxygen-free copper. This range was selected to ensure the presence of multiple grains and grain boundaries within a manageable simulation domain, enabling the capture of grain-boundary-mediated deformation mechanisms. Although the absolute grain sizes are limited, the underlying physical mechanisms and observed trends (e.g., the influence of grain boundary density on stress concentration and subsurface damage) remain qualitatively representative and provide meaningful insights for practical materials.

A single-crystal diamond tool model was built using the modeling functions embedded in LAMMPS, as illustrated in Figure 1. The diamond lattice followed a cubic diamond structure with a lattice constant of 3.5657 Å, and the clearance angle was fixed at 10°. To investigate the effects of tool geometry, three rake angles (5°, 10°, and 15°) and three tool edge radii (5 Å, 10 Å, and 15 Å) were considered, as shown in Figure 1d–i.

### 2.2. Cutting Model

**Figure 2:**
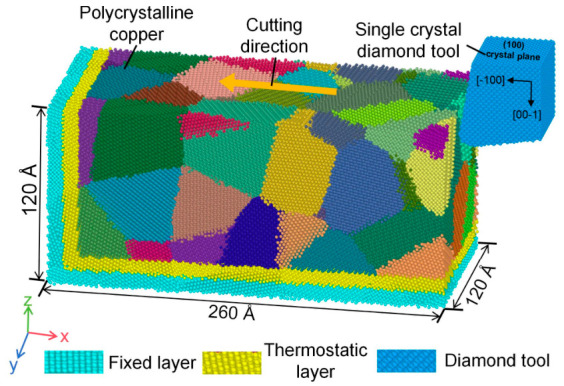
MD model of single-crystal diamond tool cutting polycrystalline copper.

An MD model of single-crystal diamond tool cutting polycrystalline copper was established, as shown in Figure 2. The simulation was conducted within a simulation box of 320 × 120 × 180 Å. The polycrystalline copper workpiece was divided into three regions: a fixed layer, a thermostatic layer, and a Newtonian layer. The fixed layer constrained the workpiece to prevent rigid-body motion during cutting, the thermostatic layer maintained thermal stability of the system, and atoms in the Newtonian layer followed Newton’s equations of motion, ensuring accurate simulation of material deformation and removal. The thicknesses of both the fixed and thermostatic layers were set to 10 Å, with the remaining region assigned as the Newtonian layer. Similarly, the diamond tool was divided into fixed, thermostatic, and Newtonian layers, with the fixed and thermostatic layers each having a thickness of 5 Å. To avoid initial interference during system relaxation, an initial separation of 5 Å along the x-direction was set between the tool and the workpiece.

### 2.3. Simulation Parameters

To investigate the effects of average grain size, cutting speed, and ambient temperature on nanometric cutting mechanisms and diamond tool wear in polycrystalline copper, simulation parameters were designed using a controlled variable approach. Average grain sizes of 34 Å, 40 Å, and 46 Å were considered. The geometric parameters of the diamond cutting tools were selected based on those of commonly used cutting tools. Tool rake angles were set to 5°, 10°, and 15°, and tool edge radii were set to 5 Å, 10 Å, and 15 Å. Ambient temperatures were set to 297 K, 497 K, 697 K, and 897 K. The upper temperature of 897 K was selected to represent severe thermal conditions that may arise locally at the tool–workpiece interface during nanometric cutting. Due to intense plastic deformation and frictional heating, the local temperature in the cutting zone can approach a significant fraction of the melting temperature of copper. Therefore, the selected temperature range aims to capture thermally activated mechanisms, including enhanced atomic mobility, local structural transition, and amorphization, rather than represent actual machining conditions.

Ren et al. [18] pointed out that the optimal range of cutting speed in MD simulation is 1–5 Å/ps when exploring the mechanism of diamond tool cutting γ-TiAl. Therefore, cutting speeds of 3 Å/ps, 4 Å/ps, 5 Å/ps, and 6 Å/ps were applied. In the present work, cutting speeds ranging from 3 to 6 Å/ps (300–600 m/s) were selected to ensure sufficient atomic deformation, stable chip formation, and observable defect evolution within a feasible simulation time. Although these values are higher than those in conventional machining, they have been widely adopted in previous MD studies to capture the essential deformation mechanisms. The focus of this work is on the relative influence of cutting speed on dislocation activity, local structural transition, and subsurface damage rather than direct reproduction of absolute industrial cutting conditions.

Prior to cutting simulations, both the polycrystalline copper workpiece and the diamond tool models were relaxed for 10,000 steps under the NVE ensemble [27] with a time step of 1 fs to ensure system stability. Detailed simulation parameters are summarized in Table 1.

### 2.4. Interatomic Potential Functions

In molecular dynamics simulations, interatomic potential functions describe the interactions between atoms, and the appropriate selection of potential functions is critical to ensuring the accuracy and reliability of simulation results. In this study, the embedded atom method (EAM) potential [28], which is widely employed for metallic systems, was adopted to describe the interactions among Cu atoms in the polycrystalline copper workpiece. The mathematical form of the EAM potential is given by Equation (1):

(1) $$E=\sum_{i}F{\left(\rho_{i}\right)}_{j}+\frac{1}{2}\sum_{i,j}\varphi_{ij}(r_{ij})$$

In Equation (1), *F*(*ρ_i_*) is the embedding energy of atom i, which is a function of the electron density (*ρ_i_*) generated by atoms other than atom i at i, *φ_ij_*(*r_ij_*) is the potential of atom i and atom j, and is a function of the truncation radius *r_ij_*.

The Tersoff potential, originally developed by Tersoff et al. [29], was employed to describe the interactions among C atoms in the single-crystal diamond cutting tool. The mathematical expression of the Tersoff potential is presented in Equation (2):

(2) $$E=\frac{1}{2}\sum_{i}\sum_{i\neq j}f_{c}(r_{ij})\left[f_{R}(r_{ij})+b_{ij}f_{A}(r_{ij})\right]$$

In Equation (2), *f_c_*(*r_ij_*) is the truncation function of the interaction between atoms, *f_R_*(*r_ij_*) is the potential of the attractive term, and *f_A_*(*r_ij_*) is the potential of the repulsive term. All three terms are functions of the truncation radius *r_ij_*. *b_ij_* is a lower-order function that includes the dependence on bond angles and multi-body interactions.

To model the interactions between C atoms in the diamond tool and Cu atoms in the workpiece, the Morse potential developed by Jia et al. [30] was employed. The mathematical form of the Morse potential is expressed in Equation (3):

(3) $$u(r_{ij})=D(e^{-2\alpha (r_{ij}-r_{0})}-2e^{-\alpha (r_{ij}-r_{0})})$$

where D is the binding energy (eV), α the elastic modulus, and *r*_0_, the equilibrium distance (nm). The values of the atomic Morse potential between Cu-C are presented in Table 2.

## 3. Atomistic Mechanism of Nanometric Cutting for the Polycrystalline Copper

### 3.1. Analysis of the Cutting Process, Mechanism and Subsurface Damage

To elucidate the material removal mechanism of polycrystalline copper at the atomic scale, the subsurface lattice evolution during nanometric cutting of polycrystalline copper with a medium grain size was analyzed using OVITO. The local atomic structures were identified using the common neighbor analysis (CNA) method, which classifies atoms into fcc, hcp, bcc, and other configurations based on their local bonding environments. Atoms that cannot be assigned to a crystalline structure are considered amorphous or disordered. It should be noted that hcp-coordinated atoms identified by CNA in fcc copper do not indicate a true fcc-to-hcp phase transformation. Instead, these atoms correspond to local defect structures such as stacking faults, deformation twins, and transient lattice distortions.

**Figure 3:**
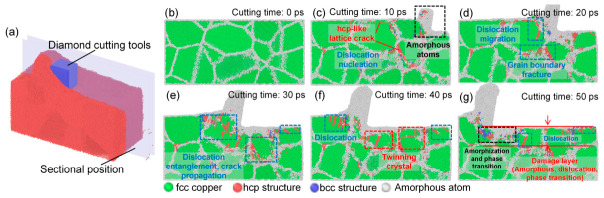
The evolution of the subsurface lattice over time during the polycrystalline copper cutting process (tool edge radius: 10 Å; tool rake angle: 10°; cutting speed: 4 Å/ps; average grain size: 40 Å). (**a**) The cross-sectional position of the subsurface (Red: polycrystalline copper, blue: diamond cutting tools), the lattice morphology of the subsurface at different cutting times: (**b**) 0 ps, (**c**) 10 ps, (**d**) 20 ps, (**e**) 30 ps, (**f**) 40 ps, and (**g**) 50 ps.

The results are shown in Figure 3. The selected observation region corresponds to a subsurface cross-section along the cutting direction (x-axis), as schematically illustrated in Figure 3a. The selected cross-section is located in the middle of the Y direction, and the thickness of the cross-section is 20 Å. The cutting subsurface positions of all polycrystalline copper involved in this study are consistent with this position. As shown in Figure 3b, prior to cutting, the subsurface grains and lattice structures of polycrystalline copper remain intact, and the Cu atoms within each grain are arranged in a well-ordered face-centered cubic structure. When the cutting time reaches 10 ps (Figure 3c), pronounced amorphization occurs within the cutting deformation zone. Meanwhile, cracks associated with hexagonal close-packed (hcp) lattice structures emerge, which indicates the nucleation and initiation of dislocations. As the cutting process continues (Figure 3d,e), the number of dislocations in the cutting deformation zone increases markedly, accompanied by dislocation multiplication and migration. Some dislocations penetrate grain boundaries, leading to grain boundary rupture. In addition, extensive dislocation entanglement and propagation of amorphous cracks are observed within the cutting deformation zone. These phenomena suggest that the dominant deformation mechanisms during nanometric cutting of polycrystalline copper are cutting-force-induced dislocation nucleation, multiplication, and migration, together with lattice structural transition and crack initiation and propagation. Further analysis of Figure 3f,g reveals that when the cutting time reaches 40 ps and 50 ps, twins are formed in both the cutting deformation zone and the subsurface region, and a high density of dislocations remains beneath the machined surface. Twinning effectively impedes dislocation motion and enhances the strength of polycrystalline copper, while dislocation entanglement further suppresses plastic deformation, thereby contributing to strain hardening during ultrahigh-speed nanometric cutting [31]. After cutting, a subsurface damaged layer remains beneath the machined surface of polycrystalline copper, characterized by atomic disorder, dislocation accumulation, and defect-related local structural changes. In this study, the subsurface damage layer is quantitatively defined as the region below the machined surface where atoms deviate from the ideal fcc structure, including atoms identified as non-fcc (e.g., hcp-like configurations and amorphous atoms) based on common neighbor analysis, together with regions exhibiting elevated atomic strain or displacement. Reducing the thickness of this layer through appropriate processing strategies is essential for improving the machining quality and surface integrity of polycrystalline copper.

**Figure 4:**
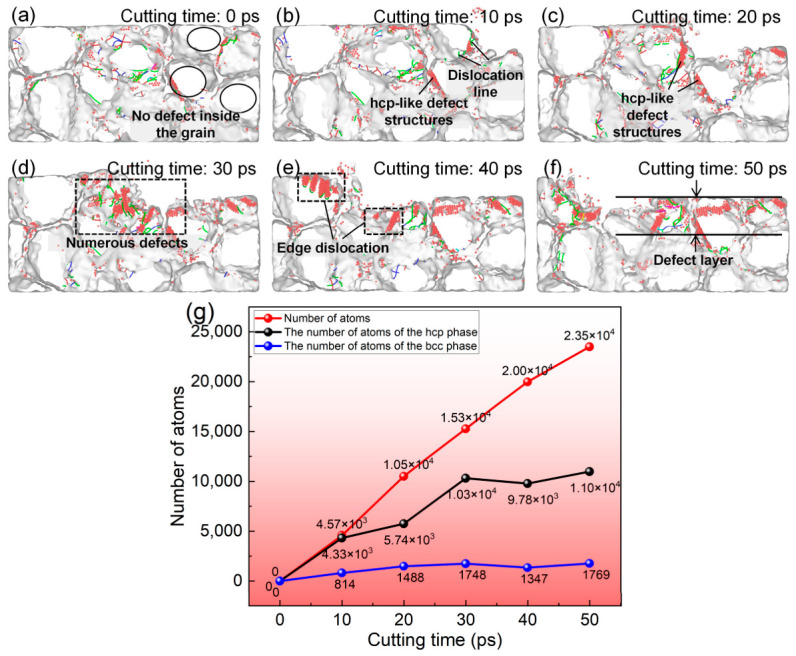
Evolution of subsurface dislocations and hcp-like defects during nanometric cutting of polycrystalline copper: subsurface structural transitions at different cutting times (tool edge radius: 10 Å; tool rake angle: 10°; cutting speed: 4 Å/ps; average grain size: 40 Å): (**a**) 0 ps, (**b**) 10 ps, (**c**) 20 ps, (**d**) 30 ps, (**e**) 40 ps, (**f**) 50 ps; (**g**) variations in the numbers of cutting-induced amorphous, hcp, and bcc atoms with cutting time.

Figure 4 illustrates the evolution of subsurface hcp-like local structures and dislocation behavior during nanometric cutting of polycrystalline copper. As shown in Figure 4a, prior to cutting, the grains within the polycrystalline copper exhibit well-defined morphologies, and no hcp-like structures, dislocations, or crack defects are observed inside the grains. When the cutting time reaches 10 ps (Figure 4b), the formation of hcp-coordinated atoms initiates within the cutting deformation zone, accompanied by the onset of dislocation multiplication. As shown in Figure 4c,d, when the cutting time increases to 20 ps and 30 ps, a large number of hcp-like defects and dislocations emerge around the cutting deformation zone. These observations confirm that the dominant material removal and deformation mechanisms during polycrystalline copper cutting are lattice structural transition and dislocation multiplication and slip induced by cutting forces. Further examination of Figure 4e,f reveals that a defective subsurface layer containing hcp-like defects and dislocation entanglement remains beneath the machined surface after cutting. The presence of such hcp-like defects within this layer can significantly deteriorate the service performance of precision-machined polycrystalline copper components. In addition, Figure 4g shows that under cutting-force-induced conditions, three distinct atomic configurations—amorphous atoms, hcp-structured atoms, and bcc-structured atoms—are generated within the polycrystalline copper during the entire cutting process. Specifically, the number of amorphous atoms increases from 0 to 23,482, the number of hcp atoms increases from 0 to 10,973, and the number of bcc atoms increases from 0 to 1769, while the number of bcc atoms remains negligible. This result demonstrates that the dominant lattice structural transitions during polycrystalline copper cutting are amorphization and the fcc → hcp-like structure, whereas the bcc phase plays a negligible role. The lattice distortion of fcc is also the main reason for dislocation initiation and expansion. Most dislocations exist in the form of an hcp-like structure.

**Figure 5:**
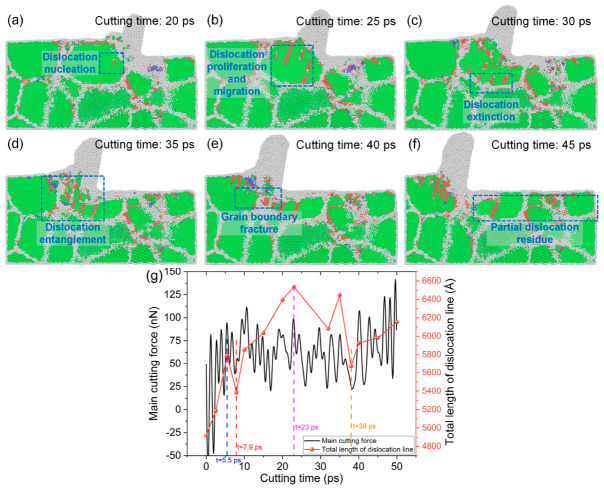
Dislocation motion in the subsurface region of the large-grain-size model during cutting: subsurface lattice morphologies at different cutting times (tool edge radius: 10 Å; tool rake angle: 10°; cutting speed: 4 Å/ps; average grain size: 46 Å): (**a**) 20 ps, (**b**) 25 ps, (**c**) 30 ps, (**d**) 35 ps, (**e**) 40 ps, (**f**) 45 ps; (**g**) variation curve of main cutting force and total length of dislocation line with time.

Dislocation nucleation, multiplication, and migration within grains are the primary mechanisms responsible for plastic deformation in polycrystalline copper. To further investigate dislocation motion mechanisms during cutting, a large-grain-size model was employed to analyze dislocation evolution in greater detail. As shown in Figure 5, when the cutting time reaches 20 ps, dislocation nucleation is observed within the grains of polycrystalline copper. At 25 ps, dislocation multiplication and migration occur at the same locations within the grains. When the cutting time increases to 30 ps, dislocations migrate toward the grain boundaries, where partial dislocation annihilation occurs. Since grain boundaries impede dislocation motion, most dislocations are annihilated at the grain boundaries. When the cutting time exceeds 35 ps, extensive dislocation entanglement appears within the cutting deformation zone. Dislocation entanglement obstructs dislocation motion, making plastic deformation more difficult and consequently increasing the cutting force. At a cutting time of 40 ps, some dislocations penetrate the grain boundaries, leading to grain boundary fracture and allowing the cutting deformation to continue. As shown in Figure 5f, after cutting, residual dislocations and twins remain in the subsurface region, forming a damaged subsurface layer. Dislocation structures were characterized using the dislocation extraction algorithm (DXA), which enables the identification of dislocation lines and the quantitative calculation of their total line length from atomic configurations. Further analysis of Figure 5g indicates that both the main cutting force–time curve and the total dislocation line length–time curve exhibit pronounced fluctuations during the cutting process. A strong positive correlation is observed between the peaks and valleys of the cutting force and those of the dislocation line length. This correlation indicates that dislocation motion is the primary cause of cutting force fluctuations: a greater total dislocation line length corresponds to a higher dislocation density and a larger cutting force, and vice versa. The MD simulation results suggest that dislocation nucleation, multiplication, and migration govern the plastic deformation of polycrystalline copper, whereas dislocation entanglement, dislocation annihilation, and grain boundary fracture are the dominant mechanisms responsible for cutting force fluctuations.

It should be noted that although the above analysis is based on a representative simulation condition, similar deformation characteristics were consistently observed across other simulation cases with different grain sizes, tool geometries, and process parameters. In particular, the formation of defect-related local structures (e.g., hcp-like configurations), dislocation activity, and subsurface damage evolution exhibit comparable trends, while their intensity and spatial distribution vary depending on the specific conditions. For example, variations in grain size mainly affect the density of grain-boundary-mediated deformation, whereas changes in tool geometry and cutting parameters influence the extent of plastic deformation and stress concentration. These comparisons indicate that the mechanisms discussed in this subsection are generally applicable, although their quantitative manifestations depend on the specific simulation conditions.

### 3.2. Effect of Grain Size on Cutting Quality and Mechanism

**Figure 6:**
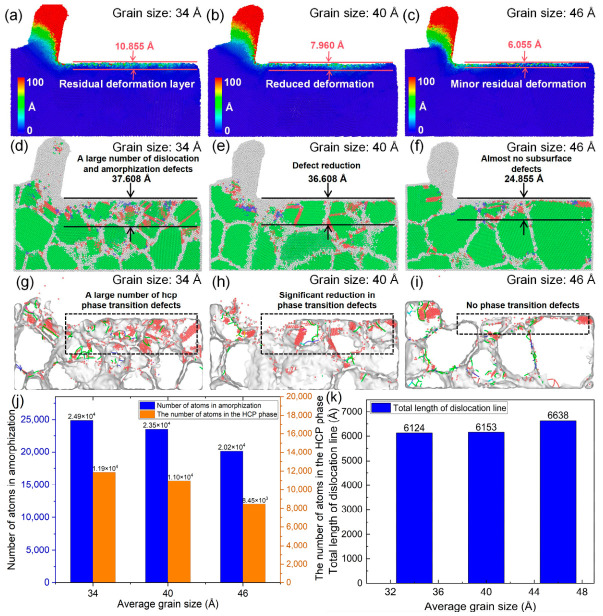
Subsurface plastic deformation, lattice morphology, and hcp-like defects of polycrystalline copper after cutting with different grain sizes (cutting speed: 4 Å/ps, tool rake angle: 10°, edge radius: 10 Å, cutting length: 140 Å). Plastic deformation under different grain sizes: (**a**) average grain size: 34 Å, (**b**) average grain size: 40 Å, (**c**) average grain size: 46 Å; subsurface lattice morphology: (**d**) average grain size: 34 Å, (**e**) average grain size: 40 Å, (**f**) average grain size: 46 Å; hcp-like defects: (**g**) average grain size: 34 Å, (**h**) average grain size: 40 Å, (**i**) average grain size: 46 Å; (**j**) variation in the number of cutting-induced amorphous and hcp atoms with grain size; (**k**) variation in total dislocation line length with grain size.

In nanometric ultra-precision machining of polycrystalline materials, grain size is a critical factor governing machining quality. To investigate the influence of grain size on cutting performance, three polycrystalline copper models with different average grain sizes were constructed. Under a cutting speed of 4 Å/ps and a cutting depth of 20 Å, the cutting results corresponding to different grain sizes are shown in Figure 6.

As shown in Figure 6a–c, with the average grain size increasing from 34 Å to 46 Å, the residual plastic deformation beneath the machined surface decreases gradually after cutting. The depth of the residual plastic deformation layer is reduced from 10.855 Å to 6.055 Å, indicating that grain size is a key factor influencing the plastic deformation behavior of polycrystalline copper during cutting. Larger grain sizes lead to less subsurface plastic deformation and, consequently, higher machining quality. As illustrated in Figure 6d, when the grain size is 34 Å, a pronounced amorphous damaged layer is observed beneath the machined surface, which contains a high density of dislocations and amorphous crack defects. Further comparison of Figure 6e,f shows that as the average grain size increases to 40 Å and 46 Å, the number of dislocations and amorphous crack defects in the subsurface region decreases significantly. Correspondingly, the depth of the residual damaged layer is reduced from 37.608 Å to 20.855 Å, and the surface flatness is markedly improved. Moreover, Figure 6g–i indicate that with increasing grain size, the number of hcp-like defects in both the cutting deformation zone and the subsurface region decreases significantly, accompanied by a pronounced reduction in the depth of the subsurface hcp-like structure layer. As shown in Figure 6j, when the material model transitions from small to large grain sizes under identical cutting conditions, the number of cutting-induced amorphous atoms decreases from 24,899 to 20,155, and the number of hcp phase atoms decreases from 11,864 to 8452. These results confirm that increasing grain size effectively suppresses amorphization damage and lattice structural transition during polycrystalline copper cutting. This behavior can be attributed to the higher density of grain boundaries in materials with smaller grain sizes. When dislocations generated in the subsurface region propagate toward grain boundaries, they are absorbed by the boundaries, leading to lattice distortion. Therefore, smaller grain sizes result in a higher density of hcp-like defects and amorphous damage in the subsurface region during cutting. Figure 6k shows that the total length of dislocation lines on the cutting subsurface increases from 6124 Å to 6638 Å with the increase in grain size. Due to the barrier and absorption effects of grain boundaries on dislocation motion, larger grain sizes correspond to a lower grain boundary density, so the total length of dislocation lines on the cutting subsurface increases with the increase in grain size.

To further clarify the effect of grain size on stress distribution within the cutting deformation zone, the von Mises stress distributions were analyzed for models with different grain sizes. Using LAMMPS software (LAMMPS 64-bit 29Aug2024-MSMPI) to calculate six stress components, including principal stresses *σ_xx_*, *σ_yy_*, *σ_zz_*, *τ_xy_*, *τ_yz_* and *τ_zx_*, for each atom in the simulation model, then calculate the von Mises stress of each atom using Equation (4) [32], and finally display the stress cloud map using OVITO software (OVITO 3.15.4).

(4) $$\sigma_{eq}=\sqrt{\frac{1}{2}\left[{\left(\sigma_{xx}-\sigma_{yy}\right)}^{2}+{\left(\sigma_{yy}-\sigma_{zz}\right)}^{2}+{\left(\sigma_{zz}-\sigma_{xx}\right)}^{2}+6(\tau_{xy}^{2}+\tau_{yz}^{2}+\tau_{zx}^{2})\right]}$$

where *σ_eq_* is the von Mises stress (unit: GPa), *σ_xx_* is the normal stress component in the x direction, *σ_yy_* is the normal stress component in the y direction, *σ_zz_* is the normal stress component in the z direction, *τ_xy_* is the shear stress parallel to the y axis in the x direction, *τ_yz_* is the shear stress parallel to the z axis in the y direction, and *τ_zx_* is the shear stress parallel to the x axis in the z direction. All six stress components can be directly calculated using LAMMPS software (LAMMPS 64-bit 29Aug2024-MSMPI). To ensure physical consistency, the atomic stress was normalized by the corresponding atomic volume, yielding a stress quantity with units of pressure. It should be noted that atomic-level stress represents a local measure derived from the virial expression and may exhibit fluctuations; therefore, it is primarily used to analyze relative variations and spatial distributions of stress during the cutting process.

**Figure 7:**
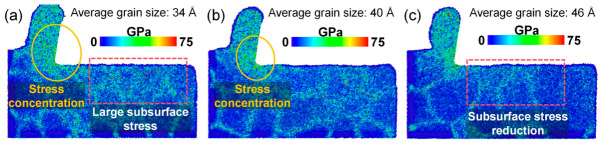
Von Mises stress distribution in the subsurface region of polycrystalline copper after cutting with different grain sizes: (**a**) average grain size: 34 Å, (**b**) average grain size: 40 Å, (**c**) average grain size: 46 Å.

As shown in Figure 7a–c, the von Mises stress within the cutting deformation zone exhibits a decreasing trend with increasing average grain size, indicating that smaller grain sizes lead to more severe stress concentration during cutting. This phenomenon is due to the tendency for stress concentration to occur at the grain boundaries within polycrystalline materials, while in fine-grained material models, the density of grain boundaries is higher, making stress concentration more likely to occur. Excessive grain boundaries therefore intensify stress localization in the cutting deformation zone of fine-grained polycrystalline copper.

**Figure 8:**
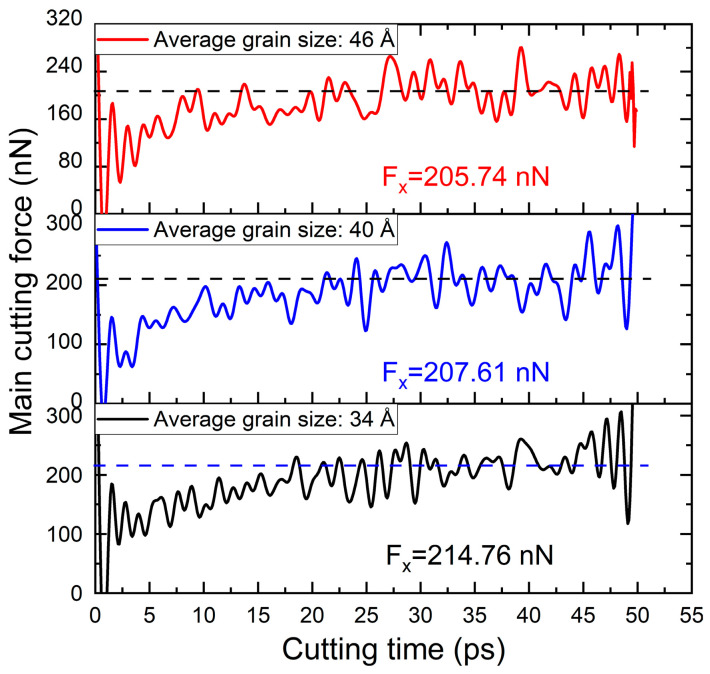
Main cutting force–time curves during polycrystalline copper cutting with different grain sizes.

Figure 8 presents the time histories of the main cutting force during polycrystalline copper cutting for different grain sizes. The average value of the stable stage in the cutting force time curve is taken as the measurement value of the cutting force. As shown in the figure, the main cutting force decreases from 214.76 nN to 205.74 nN with increasing average grain size. This trend can be attributed to differences in dislocation activity within the cutting deformation zone for different grain sizes. Previous studies have demonstrated that plastic deformation fundamentally originates from dislocation multiplication and migration [33]. When dislocation motion is impeded, the resistance to deformation increases. In small-grain models, the high density of grain boundaries significantly obstructs dislocation migration in the cutting deformation zone, resulting in greater deformation resistance and higher cutting forces. In addition, the fluctuation amplitude of the cutting force–time curve reflects the stability of the cutting process: smaller fluctuations correspond to higher process stability and better machining quality. It is evident that as grain size increases, the fluctuation amplitude of the main cutting force–time curve gradually decreases, indicating that an appropriate increase in grain size is beneficial for improving cutting stability.

It is worth noting that the significant force fluctuations observed at the initial and final stages in Figure 8 are intrinsic to the MD simulation of nanometric cutting. The initial surge in cutting force is attributed to the abrupt contact between the diamond tool and the polycrystalline copper, representing the transition from elastic compression to the burst of dislocation nucleation. As shown in Figure 5g, the force peaks are highly synchronized with the rapid increase in total dislocation length. The fluctuations at the end of the simulation are primarily due to the release of accumulated strain energy and the dynamic redistribution of atoms as the tool concludes its motion. These variations are consistent with the atomic-scale plastic deformation mechanisms discussed in Section 3.1.

## 4. Effect of Tool Geometric Parameters

Numerous studies have demonstrated that the tool rake angle and edge radius are critical factors influencing cutting performance and surface integrity in precision machining [34,35].

### 4.1. Influence of Tool Rake Angle on Cutting Performance

**Figure 9:**
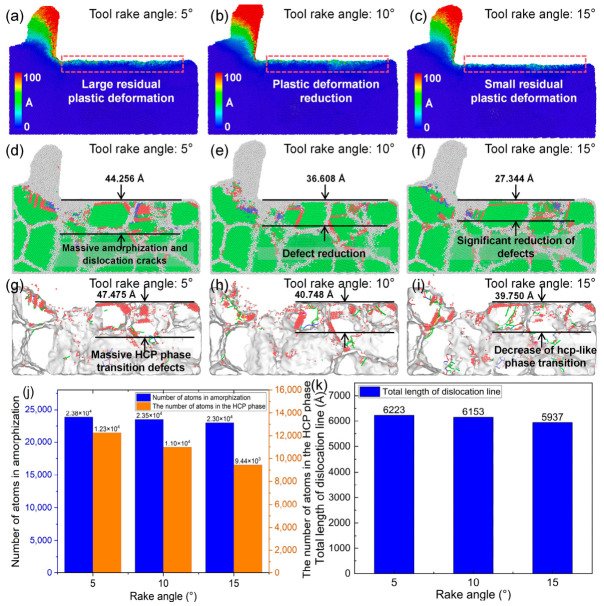
(**a**–**i**) Subsurface plastic deformation, lattice morphology, and hcp-like defects in polycrystalline copper after cutting with different tool rake angles. (**j**) Variation in the number of cutting-induced amorphous and hcp atoms with rake angle. (**k**) Variation in total dislocation line length beneath the machined surface with rake angle (tool edge radius: 10 Å; cutting speed: 4 Å/ps; average grain size: 40 Å; cutting length: 140 Å).

Figure 9 presents the effects of different tool rake angles on the cutting behavior of polycrystalline copper under identical cutting and material parameters. As shown in Figure 9a–c, increasing the tool rake angle results in a pronounced reduction in both the residual plastic deformation beneath the machined surface and the plastic deformation within the cutting deformation zone, accompanied by a decrease in chip width. This result implies that an appropriate increase in rake angle not only suppresses subsurface residual deformation but also facilitates chip evacuation, thereby effectively improving cutting quality. As illustrated in Figure 9d–f, when the rake angle increases from 5° to 15°, the number of amorphous cracks and dislocations in both the cutting deformation zone and the subsurface region decreases, and the depth of the damaged layer is reduced from 44.256 Å to 27.344 Å. Furthermore, Figure 9g–i show that the number of hcp-like defects in the subsurface region decreases significantly with increasing rake angle, and the depth of the defect layer containing pronounced hcp-like defects is reduced from 47.475 Å to 39.750 Å. Quantitative analysis in Figure 9j,k reveal that as the rake angle increases from 5° to 15°, the number of cutting-induced amorphous atoms decreases from 23,843 to 22,973, the number of hcp phase atoms decreases from 12,255 to 9440, and the total dislocation line length decreases from 6223 Å to 5937 Å. The MD simulation results indicate that, within an appropriate range, increasing the tool rake angle can effectively reduce the compressive-induced plastic deformation in the cutting deformation zone. As a result, local structural transition, amorphization damage, and residual plastic deformation in the subsurface are significantly suppressed, leading to an overall improvement in machining quality.

### 4.2. Influence of Tool Edge Radius on Cutting Performance

**Figure 10:**
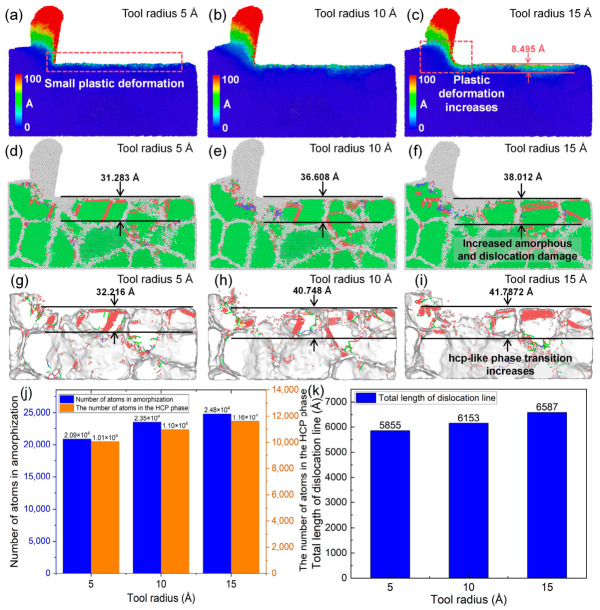
(**a**–**i**) Subsurface plastic deformation, lattice morphology, and hcp-like defects in polycrystalline copper after cutting with different tool edge radii. (**j**) Variation in the number of cutting-induced amorphous and hcp atoms with tool edge radius. (**k**) Variation in total dislocation line length beneath the machined surface with tool edge radius (tool rake angle: 10°; cutting speed: 4 Å/ps; average grain size: 40 Å; cutting length: 140 Å).

Figure 10 illustrates the effects of different tool edge radii on the cutting behavior of polycrystalline copper under identical cutting and material conditions. As shown by the atomic displacement contours in Figure 10a–c, the tool edge radius has a significant influence on atomic displacement mechanisms within the cutting deformation zone. When the edge radius is 5 Å, atomic displacement within the cutting deformation zone is relatively small, the machined surface remains smooth, and no deep residual plastic deformation layer is observed beneath the surface. When the edge radius increases to 10 Å, both atomic displacement in the cutting deformation zone and residual plastic deformation beneath the surface increase. When the edge radius reaches 15 Å, a residual plastic deformation layer with a depth of approximately 8.495 Å remains beneath the machined surface. These results indicate that the tool edge radius is a key factor affecting the machining quality of polycrystalline copper, with smaller edge radii generally leading to higher machining quality. Figure 10d–i further show the subsurface lattice morphology and lattice structural transition and dislocation defects under different edge radii. With increasing edge radius, subsurface amorphization damage, dislocation accumulation, and hcp-like defects increase markedly. As shown in Figure 10j,k, when the edge radius increases from 5 Å to 15 Å, the number of hcp atoms beneath the machined surface increases from 10,056 to 11,606, and the total dislocation line length increases from 5885 Å to 6587 Å. These MD results confirm that increasing the tool edge radius intensifies subsurface dislocation activity, amorphization, and lattice structural transition, thereby degrading cutting quality.

### 4.3. Influence of Tool Rake Angle and Tool Edge Radius on Tool Damage

**Figure 11:**
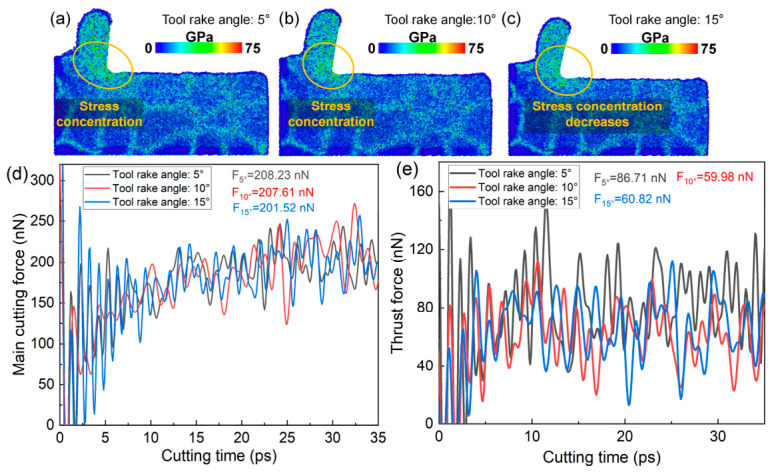
(**a**–**c**) Von Mises stress on the subsurface of polycrystalline copper cutting under different tool rake angles (cutting time: 35 ps). (**d**) Main cutting force–time curves and (**e**) thrust force–time curves during polycrystalline copper cutting with different tool rake angles.

The above analysis indicates that the tool rake angle has a significant influence on the plastic deformation behavior within the cutting deformation zone of polycrystalline copper. It is therefore expected to also affect the cutting forces and the stress distribution in this region. Figure 11a–c present the distribution of von Mises stress within the cutting deformation zone at the same cutting instant under different tool rake angles. As the tool rake angle increases, the stress concentration in the deformation zone is alleviated, accompanied by a reduction in the overall stress magnitude. This suggests that increasing the rake angle can effectively mitigate stress concentration within the cutting deformation zone. Figure 11d,e illustrate the temporal evolution of the principal cutting force and thrust force under different rake angles. The average value during the steady-state stage of the force–time curve is taken as the representative cutting force. When the rake angle increases from 5° to 15°, the principal cutting force exhibits no significant variation, while the thrust force decreases from 86.71 nN to 60.82 nN. In addition, as shown in Figure 9k, the total length of dislocation lines in the subsurface region decreases with increasing rake angle. The MD simulation results suggest that an appropriate increase in the tool rake angle can reduce dislocation density, thereby alleviating stress concentration in the cutting deformation zone and lowering cutting forces. This, in turn, decreases the likelihood of tool damage and machining defects induced by excessive stress and cutting forces.

**Figure 12:**
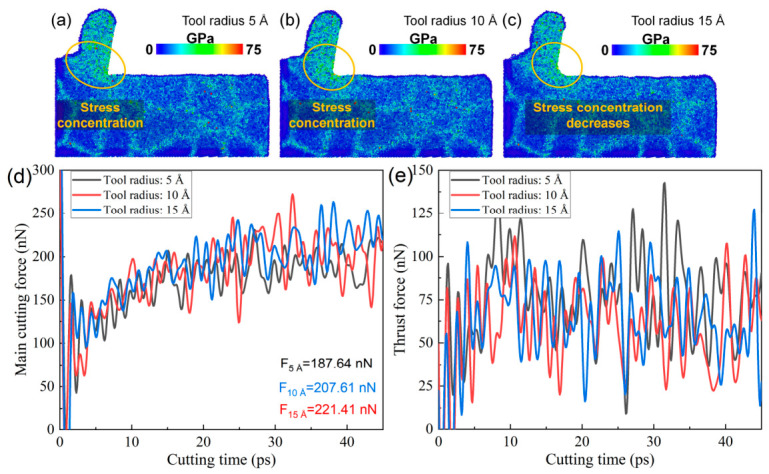
(**a**–**c**) Von Mises stress on the subsurface of polycrystalline copper cutting under different edge radii (cutting time: 35 ps). (**d**) Main cutting force–time curves and (**e**) thrust force–time curves during polycrystalline copper cutting with different tool edge radii.

Since the tool edge radius strongly influences plastic deformation within the cutting deformation zone, it is expected to have a significant impact on the cutting force and the stress distribution in the cutting deformation zone. Figure 12a–c show the variation in von Mises stress in the cutting deformation zone of polycrystalline copper at the same cutting instant with different tool edge radii. It is observed that, as the edge radius increases, the stress level in the cutting zone decreases and the degree of stress concentration is significantly alleviated. This suggests that increasing the radius of the tool edge can increase the contact area between the tool tip and polycrystalline copper material, reduce the material extrusion effect in the cutting deformation zone, and thus reduce the stress concentration in the cutting deformation zone. Figure 12d,e show the time histories of the main cutting force and thrust force during diamond cutting of polycrystalline copper with different edge radii. As the edge radius increases from 5 Å to 15 Å, the main cutting force increases from 187.64 nN to 221.41 nN, and there is no significant change in the force of the thrust force under identical cutting conditions, indicating that a larger edge radius leads to greater cutting resistance. Combined with Figure 10k, it is evident that the total dislocation line length beneath the machined surface increases at all cutting times as the edge radius increases. The MD simulation results indicate that a larger edge radius increases the dislocation density within the cutting deformation zone. Excessively high dislocation densities impede dislocation motion, hinder plastic deformation, and consequently result in higher cutting forces.

**Figure 13:**
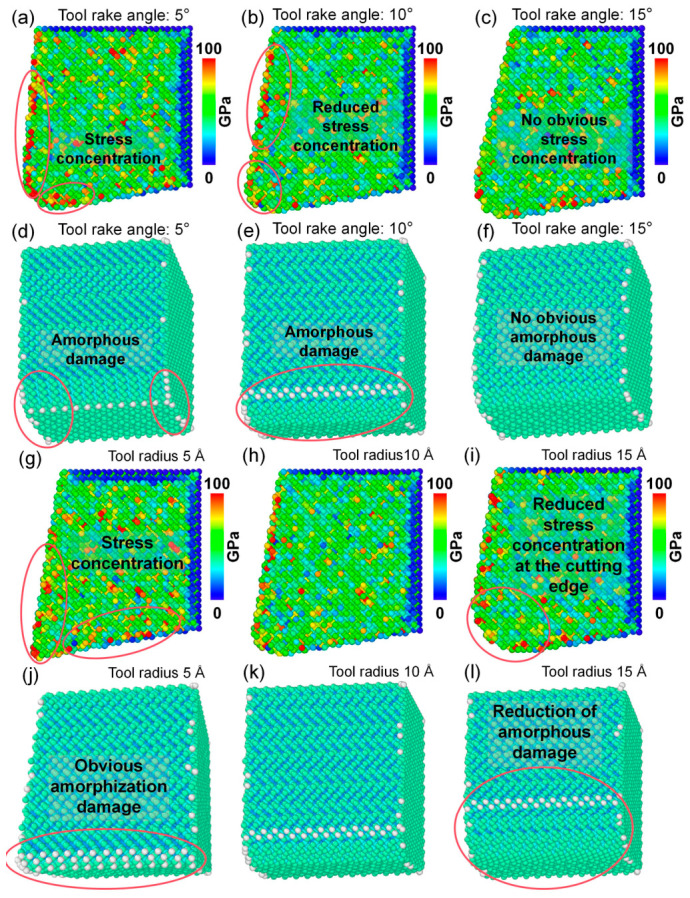
(**a**–**c**) Von Mises stress distributions inside the tool after cutting with different rake angles; (**d**–**f**) lattice morphologies of the tool after cutting with different rake angles; (**g**–**i**) von Mises stress distributions inside the tool after cutting with different edge radii; (**j**–**l**) lattice morphologies of the tool after cutting with different edge radii (cutting speed: 4 Å/ps; average grain size: 40 Å).

Although increasing the tool rake angle and reducing the tool edge radius within an appropriate range can effectively reduce subsurface damage and improve machining quality, changes in tool geometry also influence the stress state and wear behavior of the tool during repeated cutting. As shown in Figure 13a–c, when the rake angle increases from 5° to 15°, the von Mises stress within the diamond tool at the same cutting time exhibits a decreasing trend, indicating that increasing the rake angle within a certain range alleviates stress concentration within the tool. Figure 13d–f further show that as the rake angle increases from 5° to 15°, the number of amorphous atoms on the diamond tool surface after cutting decreases from 57 to 39, demonstrating that an appropriate increase in rake angle can mitigate tool wear. As shown in Figure 13g–i, when the tool edge radius increases from 5 Å to 15 Å, the von Mises stress within the tool gradually decreases. An excessively small edge radius leads to severe stress concentration at the tool tip. Correspondingly, Figure 13j–l show that as the tool edge radius increases, the number of amorphous atoms on the diamond tool surface after cutting decreases from 87 to 60, confirming that an excessively small edge radius accelerates tool wear. Overall, the MD simulation results suggest that increasing the tool rake angle within an appropriate range not only improves machining quality but also reduces tool wear. Although reducing the tool edge radius can enhance cutting quality, an excessively small edge radius intensifies stress concentration within the tool and accelerates tool wear. According to Ref. [36], Cui et al. experimentally demonstrated, through precision diamond cutting of polycrystalline copper, that excessive internal stress at the tool edge accelerates edge wear, which is consistent with the present findings regarding the effect of stress on tool wear. Additionally, Liu et al. [37] reported that appropriately increasing the tool rake angle and edge radius can reduce the wear of diamond tool edges to a certain extent, which is also in agreement with the MD simulation results of this study. Based on these comparisons with existing literature, the MD simulation results concerning the mechanism of tool wear in this work are considered to be reliable.

Tool wear in this study is evaluated based on the number of amorphous atoms within the diamond tool. This indicator reflects the degree of local structural disorder and bond breakage induced by severe stress, friction, and thermal effects during cutting. An increase in amorphous atoms is therefore associated with the degradation of the crystalline diamond structure at the atomic scale. However, it should be noted that this metric primarily reflects local structural disorder and does not fully represent all aspects of tool wear observed in practical machining, such as material loss, graphitization, or large-scale fracture. Therefore, the present evaluation is intended to provide a qualitative and comparative measure of tool degradation under different cutting conditions rather than an absolute quantification of wear.

In conclusion, the tool edge radius exerts a dominant influence in determining both surface integrity and tool durability. A smaller edge radius reduces plowing effects and subsurface damage, thereby improving surface quality. However, it also leads to higher stress concentration and intensified atomic-scale interaction at the tool–workpiece interface, which accelerates structural degradation of the diamond tool, as evidenced by the increased formation of amorphous atoms. In contrast, a larger edge radius can alleviate local stress concentration on the tool and mitigate wear to some extent, but it simultaneously enhances plowing and subsurface deformation, resulting in poorer surface integrity. These results indicate a clear trade-off between achieving low subsurface damage and minimizing tool wear. Therefore, an appropriate selection of tool edge radius is required to balance machining quality and tool life in nanometric cutting of polycrystalline copper.

## 5. Effects of Process Parameters

### 5.1. Influence of Cutting Speed on Cutting Performance and Tool Damage

**Figure 14:**
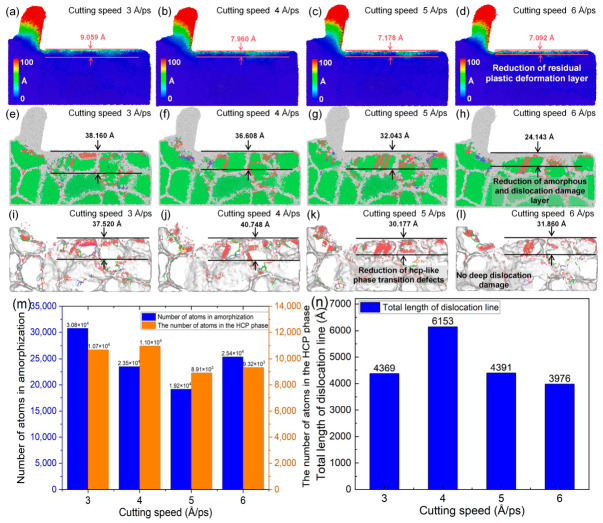
(**a**–**l**) Subsurface plastic deformation, lattice morphology, and phase-transformation defects of polycrystalline copper after cutting at different cutting speeds. (**m**) Variation in cutting-induced amorphous and hcp phase atom numbers with cutting speed. (**n**) Variation in the total dislocation line length beneath the machined surface with cutting speed (tool rake angle: 10°; edge radius: 10 Å; average grain size: 40 Å; cutting length: 140 Å).

In nanoscale ultra-precision machining, the cutting speed is a critical parameter that significantly influences the machining quality. Figure 14 illustrates the nanoscale cutting behavior of polycrystalline copper under different cutting speeds while all other conditions remain unchanged. As shown in Figure 14a,e, when the cutting speed is 3 Å/ps, an evident residual plastic deformation layer is observed beneath the machined surface, accompanied by a large number of amorphous regions and phase-transformation defects within the subsurface lattice structure. By comparing Figure 14b–d and Figure 14f–h, it can be observed that the depth of the residual plastic deformation layer beneath the machined surface decreases from 9.059 Å to 7.092 Å as the cutting speed increases gradually to 6 Å/ps. Meanwhile, the depth of the amorphization- and dislocation-dominated damage layer within the subsurface lattice is reduced to 24.143 Å. Further analysis of Figure 14i–l indicates that with increasing cutting speed up to 6 Å/ps, the depth of the subsurface hcp-like structure damage layer decreases from 37.520 Å to 31.860 Å, accompanied by a significant reduction in phase-transformation defects. As shown in Figure 14m,n, the number of cutting-induced amorphous atoms decreased from 30,766 to 19,166 when the cutting speed increased from 3 Å/ps to 6 Å/ps and then increased to 25,393; the number of hcp phase atoms decreased from 10,685 to 9322. The total dislocation line length first increases from 4369 Å to 6153 Å and then gradually decreases to 3736 Å. Detailed analysis of Figure 13 reveals a non-monotonic trend in both amorphous atom count and dislocation length with increasing cutting speed. The initial decrease in amorphous atoms, together with an increase in dislocation length, indicates that moderate cutting speeds promote energy dissipation through dislocation-mediated plasticity rather than through atomic disorder accumulation. However, as the cutting speed exceeds a critical threshold, the total dislocation length begins to decrease while the number of amorphous atoms significantly rises. This transition is attributed to the combined effects of high strain rates and cutting heat. At extreme speeds, the extremely short interaction time suppresses the formation and propagation of dislocation loops (kinematic constraint), while the intensified mechanical impact and localized temperature rise force the lattice into a highly disordered state, enhancing the mechanical amorphization process. These results indicate a shift in the dominant material removal mechanism from dislocation-based plasticity to amorphization-dominated deformation at ultra-high cutting speeds.

In addition, as shown in Figure 14l, under ultra-high-speed cutting conditions, both dislocation lines and HCP-phase defects are mainly concentrated near the machined surface, while almost no dislocations are observed in the deeper subsurface region. This phenomenon is commonly referred to as the “skin effect” [38]. The MD simulation results indicate that at extremely high strain rates associated with ultra-high-speed cutting, dislocation motion in the subsurface is suppressed, resulting in a reduction in the total dislocation length and a concomitant decrease in subsurface plastic deformation and local structural transition damage.

**Figure 15:**
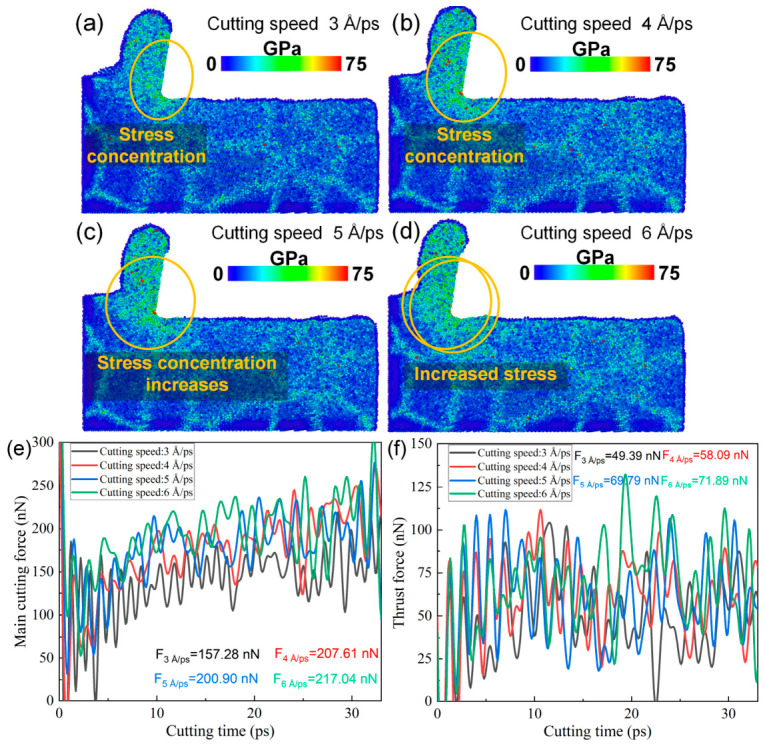
(**a**–**d**) Von Mises stress distributions in the subsurface of polycrystalline copper at different cutting speeds (cutting length: 140 Å); (**e**) time histories of the main cutting force at different cutting speeds; and (**f**) time histories of the thrust force at different cutting speeds.

From the above analysis, it can be concluded that increasing the cutting speed suppresses dislocation-mediated plastic deformation in the cutting deformation zone of polycrystalline copper, indicating a change in the dominant deformation mechanisms under high-speed cutting conditions. Since internal stress plays a key role in governing deformation behavior, the evolution of the von Mises stress in the cutting deformation zone with cutting speed was further analyzed. As shown in Figure 15a–d, when the cutting speed increases from 3 to 6 Å/ps, the von Mises stress in the deformation zone increases markedly, accompanied by more pronounced stress concentration. The elevated stress levels, together with reduced dislocation activity, promote localized atomic disorder and may increase the tendency for non-uniform deformation. Therefore, increasing the cutting speed intensifies the stress concentration in the cutting deformation zone and alters the deformation mode. This shift contributes to reduced residual plastic deformation in certain regions, which may be beneficial for improving surface integrity under specific conditions. The time histories of the main cutting force and the thrust force are shown in Figure 15e,f. With increasing cutting speed, the main cutting force increases from 157.28 nN to 217.04 nN, and the thrust force exhibits a similar upward trend. This suggests that higher cutting speeds lead to elevated stress in the cutting deformation zone, thereby increasing the cutting resistance and resulting in larger cutting forces.

**Figure 16:**
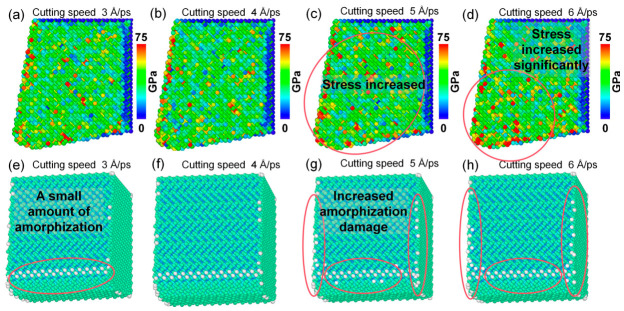
(**a**–**d**) Von Mises stress distributions of the diamond tool after cutting at different cutting speeds. (**e**–**h**) Lattice morphologies of the diamond tool after cutting at different cutting speeds (cutting length: 140 Å).

Excessively high cutting speeds may induce tool damage. Therefore, the stress distribution and lattice morphology of the diamond tool after cutting at different speeds were further analyzed, as shown in Figure 16. As illustrated in Figure 16a–h, the von Mises stress values inside and at the cutting edge of diamond cutting tools significantly increase when the cutting speed increases from 3 Å/ps to 6 Å/ps, and the number of amorphous atoms on the tool surface rises from 57 to 94. The results indicate that cutting speed also affects tool integrity, with higher cutting speeds leading to increased stress concentration and accelerated tool wear. Although increasing the cutting speed can effectively reduce the cutting subsurface damage, a too-high cutting speed can easily cause a too-high cutting force, which will lead to the aggravation of tool wear. Therefore, the cutting speed should be reasonably controlled in the actual processing so as to improve the cutting quality and avoid tool wear.

### 5.2. Influence of Ambient Temperature on Cutting Performance and Tool Damage

In practical precision cutting of polycrystalline copper, different machining conditions lead to varying cutting temperatures within the deformation zone. Temperature is a crucial factor influencing atomic thermal motion and dislocation activity in polycrystalline copper. To investigate its effect on the nanoscale cutting mechanism, the ambient temperature of the simulation system was varied.

**Figure 17:**
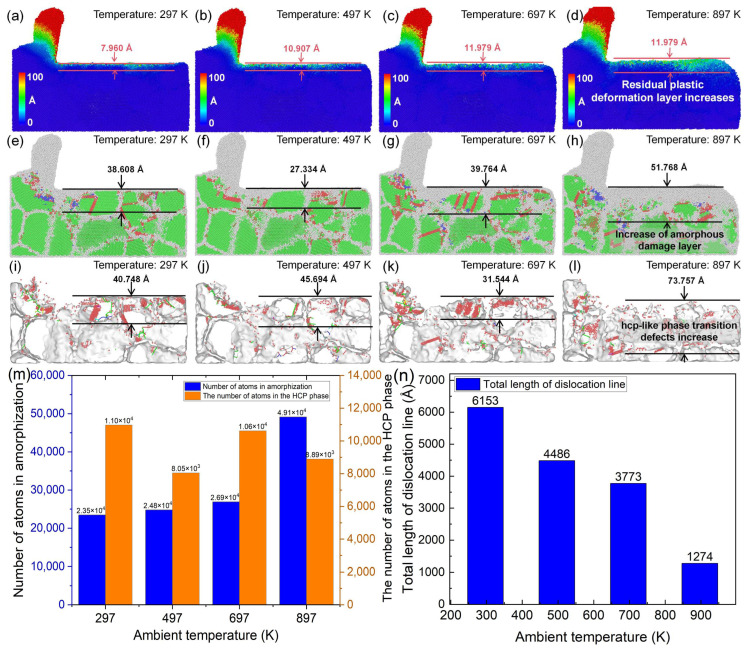
(**a**–**l**) Subsurface plastic deformation, lattice morphology, and phase-transformation defects of polycrystalline copper after cutting at different ambient temperatures. (**m**) Variation in cutting-induced amorphous and hcp phase atom numbers with ambient temperature. (**n**) Variation in total dislocation line length beneath the machined surface with ambient temperature (tool rake angle: 10°; edge radius: 10 Å; cutting speed: 4 Å/ps; average grain size: 40 Å; cutting length: 140 Å).

As shown in Figure 17a–d, when the ambient temperature increases from 297 K to 897 K, the atomic displacement deformation in both the cutting deformation zone and the subsurface region exhibits an increasing trend. The thickness of the residual plastic deformation layer beneath the machined surface increases from 7.960 Å to 11.979 Å, indicating that higher temperatures intensify plastic deformation during cutting and deteriorate surface quality. Further analysis of Figure 17e–l reveals that the amorphous cracks, hcp-like defects, and dislocation line density in the subsurface region increase with increasing ambient temperature. The damage layer depth increases from 36.608 Å to 51.768 Å, and the phase defect layer depth decreases from 40.748 Å to 73.757 Å. As shown in Figure 17m, when the ambient temperature increases from 297 K to 897 K, the number of cutting-induced amorphous atoms increases from 23,482 to 49,125, while the number of hcp phase atoms decreases from 10,967 to 8892. As shown in Figure 17n, the constant reduction in the total dislocation length with increasing temperature is primarily attributed to the enhanced thermal recovery process. High temperatures increase the kinetic energy of atoms, facilitating the annihilation of dislocations and the absorption of dislocation loops by grain boundaries. Concurrently, the material undergoes significant thermal softening, which lowers the critical stress for lattice disordering. At the highest temperature investigated, the dramatic increase in the number of amorphous atoms indicates a transition in the deformation mode. Specifically, the lattice stability is compromised by intense thermal vibrations, allowing the mechanical energy of the tool to be dissipated through extensive amorphization rather than discrete dislocation propagation. This leads to a shallower dislocation-affected zone but a more pronounced amorphous damaged layer at extreme temperatures. Therefore, in practical ultra-precision cutting of polycrystalline copper, effective measures should be adopted to suppress excessive cutting temperatures in order to improve machining quality.

**Figure 18:**
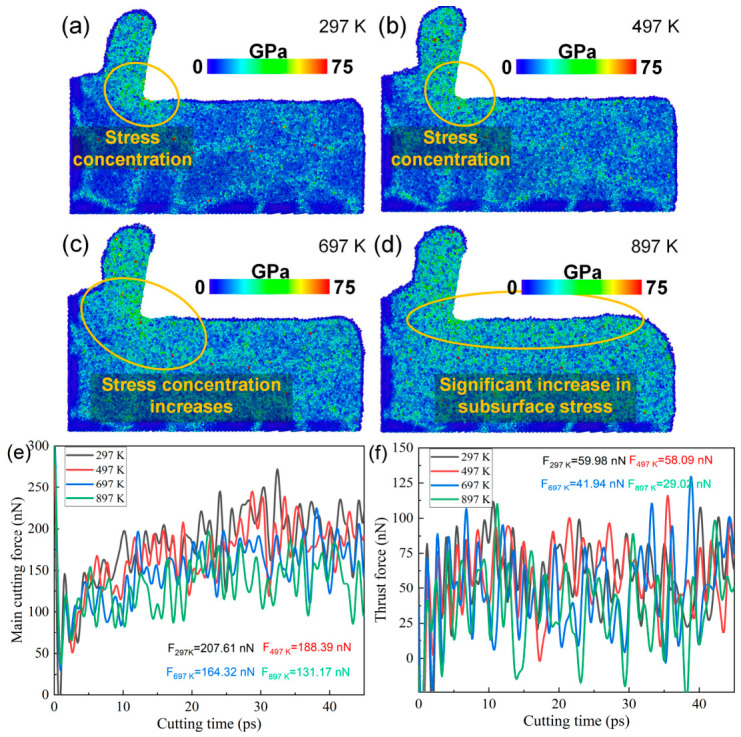
(**a**–**d**) Von Mises stress distributions in the subsurface of polycrystalline copper at different ambient temperatures (cutting length: 140 Å). (**e**) Main cutting force–time curves at different ambient temperatures. (**f**) Thrust force–time curves at different ambient temperatures.

The distributions of von Mises stress in the cutting deformation zone of polycrystalline copper under different ambient temperatures are shown in Figure 18a–d. It can be observed that both the stress magnitude and the degree of stress concentration in the cutting deformation zone increase significantly with rising ambient temperature. The cutting force responses at different ambient temperatures are presented in Figure 18e,f. It can be observed that both the main cutting force and thrust force decrease as the ambient temperature increases from 297 K to 897 K. Combined with Figure 17n, it is evident that the total dislocation line length beneath the machined surface decreases at the same cutting time with increasing temperature. These MD simulation results indicate that higher ambient temperatures reduce dislocation density, which facilitates dislocation motion within polycrystalline copper and promotes plastic deformation, thereby leading to lower cutting forces. The reduction in dislocation density at elevated temperatures can be attributed to enhanced atomic thermal motion and lattice deformation, which facilitate dislocation annihilation and recovery. In addition, increasing the ambient temperature reduces the dislocation density, while the stress in the cutting deformation zone increases. The reason for the stress increase here is not dislocation, but the intensification of atomic thermal motion.

**Figure 19:**
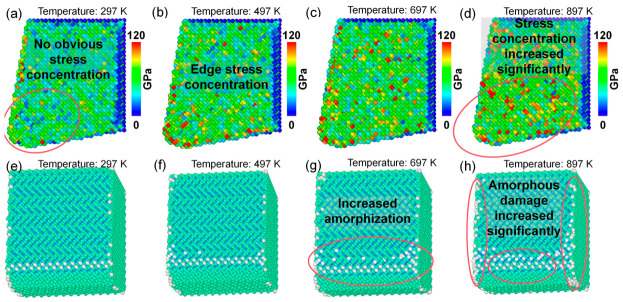
(**a**–**d**) Von Mises stress distributions of the diamond tool after cutting at different ambient temperatures. (**e**–**h**) Lattice morphologies of the diamond tool surface after cutting at different ambient temperatures (cutting length: 140 Å).

Cutting temperature rise is a key factor influencing diamond tool wear. Therefore, the stress distribution and lattice morphology of single-crystal diamond tools under different ambient temperatures were analyzed, as shown in Figure 18. As illustrated in Figure 19a–d, when the ambient temperature increases from 297 K to 897 K, the von Mises stress near the cutting edge of the diamond tool increases progressively, indicating more severe stress concentration at higher temperatures. Further observation of Figure 19e–h reveals that the number of amorphous carbon atoms generated on the diamond tool surface during cutting increases from 59 to 151 with increasing temperature, demonstrating that elevated temperatures promote amorphization damage on the diamond tool surface. These MD simulation results confirm that cutting temperature rise is a critical factor governing diamond tool wear, and higher temperatures inevitably lead to more severe tool damage.

### 5.3. Summary of the Effects of Key Parameters

The effects of key parameters on subsurface damage, cutting forces, stress distribution, and tool wear are systematically summarized as follows.

Grain size exerts a dominant influence in governing deformation heterogeneity: increasing grain size reduces grain boundary density, thereby alleviating stress concentration and suppressing subsurface amorphization and lattice structural transition, which ultimately improves surface integrity and reduces cutting force fluctuations. Tool geometry significantly influences material removal behavior. A larger rake angle facilitates chip flow and reduces cutting resistance, leading to lower subsurface damage, whereas an increased tool edge radius enhances plowing effects, resulting in higher cutting forces, intensified dislocation activity, and more severe lattice structural transition and subsurface defects.

Cutting speed exhibits a dual effect: higher speeds suppress dislocation density and lattice distortion due to limited time for defect nucleation and propagation, thereby reducing subsurface damage; however, they also induce higher stress concentration and thermal effects at the tool-workpiece interface, which can aggravate tool wear. Ambient temperature strongly affects atomic mobility and deformation mechanisms. Elevated temperature promotes atomic diffusion and disorder, leading to increased amorphization and deeper plastic deformation, while simultaneously facilitating dislocation annihilation and reducing dislocation density. In addition, higher temperatures accelerate tool degradation due to enhanced adhesion and atomic-scale wear processes.

Overall, the subsurface damage and tool wear in nanometric cutting of polycrystalline copper are governed by the coupled effects of microstructural characteristics, tool geometry, and process parameters. Optimizing these factors is essential for achieving low-damage machining and extended tool life.

## 6. Conclusions

In this study, molecular dynamics simulations were employed to investigate the nanoscale cutting behavior of polycrystalline copper from an atomic perspective, with emphasis on lattice evolution and material removal mechanisms. The effects of grain size, tool geometry, and cutting parameters on atomic migration, structural transition, and dislocation activity were systematically analyzed. The main conclusions are summarized as follows:

(1) Nanoscale material removal in polycrystalline copper is governed by cutting-induced lattice structural transition accompanied by mechanical amorphization. Dislocation nucleation, motion, and interaction dominate plastic deformation, while dislocation entanglement and annihilation contribute to cutting force fluctuations. A positive correlation exists between cutting force and dislocation density, and a damaged subsurface layer containing hcp-like defects and residual plastic deformation remains after machining.

(2) Grain size exerts a dominant influence in cutting behavior. Increasing grain size reduces grain boundary density, facilitating dislocation motion and alleviating stress concentration, thereby suppressing subsurface amorphization and lattice distortion. Due to computational limitations, these results mainly capture qualitative trends due to computational limitations.

(3) Tool geometry significantly affects cutting performance. Increasing rake angle promotes chip flow and reduces plastic deformation, leading to lower subsurface damage and improved surface integrity. In contrast, a larger edge radius enhances plowing-dominated deformation, increasing dislocation density, cutting forces, and subsurface damage. An appropriate combination of rake angle and edge radius is therefore essential, considering the trade-off with tool wear.

(4) Cutting speed and ambient temperature strongly influence deformation behavior. Higher cutting speeds suppress dislocation activity and reduce subsurface damage but increase stress concentration and tool degradation. Elevated temperatures enhance atomic mobility, promoting amorphization and defect formation, which may deteriorate surface integrity.

(5) Compared with previous MD studies that primarily focus on individual parameters, this work provides a more integrated analysis by considering the combined effects of grain size, tool geometry, cutting speed, and temperature. The results establish correlations between atomic-scale features (e.g., defect evolution and amorphization) and macroscopic responses such as subsurface damage, cutting forces, and tool wear. These findings offer additional insight into the nanometric cutting behavior of polycrystalline copper.

(6) It should be noted that the findings of this study are derived from molecular dynamics simulations conducted under specific conditions. As such, further experimental validation and multiscale investigations are necessary to extend the applicability and generality of these conclusions. In addition, the inherent limitations of molecular dynamics, including the relatively small system size and short simulation time scales, constrain the present results to primarily qualitative insights. Future work integrating larger-scale simulations with experimental studies will be essential to achieve more comprehensive validation and deeper understanding.

## Figures and Tables

**Table 1:** Simulation parameter settings.

Material Properties	Crystal Type	Size	Number of Atoms	Lattice Structure
Diamond cutting tools	Single-crystal	55 Å × 50 Å × 60 Å;	C atoms: 23,702	cubic
Polycrystalline copper	Polycrystalline	260 Å × 120 Å × 120 Å	Cu atoms: 316,898	fcc
Potential function	Cu-Cu: EAM; Cu-C: Morse; C-C: Tersoff.			
Time step	1 fs			
Cutting length	20 nm			
Cutting depth	20 Å			
Average grain size:	34 Å, 40 Å, and 46 Å			
Rake angle of the tool	5°, 10°, 15°			
Tool edge radius	5 Å, 10 Å, 15 Å,			
Cutting speed	3 Å/ps, 4 Å/ps, 5 Å/ps, 6 Å/ps			
Temperature	297 K, 497 K, 697 K, 897 K			

**Table 2:** Parameter table of Morse potential function.

	α (eV)	D (1/Å)	r_0_ (Å)
Cu-C	0.087	5.14	2.05

## Data Availability

Data underlying the results presented in this paper are not publicly available at this time but may be obtained from the authors upon reasonable request.
